# In vitro studies data on anticancer activity of *Caesalpinia sappan L.* heartwood and leaf extracts on *MCF7* and *A549* cell lines

**DOI:** 10.1016/j.dib.2018.05.050

**Published:** 2018-05-21

**Authors:** Arunkumar Naik Bukke, Fathima Nazneen Hadi, K. Suresh Babu, P. Chandramati shankar

**Affiliations:** a Department of Biotechnology, Yogi Vemana University, Kadapa, Andhra Pradesh, India; b Natural Products Laboratory, Division of Natural Products Chemistry, CSIR-Indian Institute of Chemical Technology, Hyderabad 500007, India

**Keywords:** Chloroform extract, Methanol extract, Water extract, *Caesalpinia sappan L.*, Cytotoxicity, *MCF7*, *A549*, MTT Assay

## Abstract

This article contains data on in vitro cytotoxicity activity of chloroform, methanolic and water extracts of leaf and heartwood of *Caesalpinia sappan L.* a medicinal plant against Breast cancer (MCF-7) and Lung cancer (A-549) cells. This data shows that Brazilin A, a natural bioactive compound in heartwood of *Caesalpinia sappan L.*induced cell death in breast cancer (MCF-7) cells. The therapeutic property was further proved by docking the Brazilin A molecule against BCL-2 protein (an apoptotic inhibitor) using auto dock tools.

**Specifications table**

**Table:** 

Subject area	Biology
More specific subject area	Screening for Anti Cancer Activity in medicinal plants and Ethno medicines
Type of data	Tables, microscopy images, text file, graphs, Chromatogram figure, docking images
How data was acquired	Conducting of anticancer activity assays and cytotoxicity studies with methanol and water extracts of leaf and heartwood *of Caesalpinia sappan* L. on MCF-7 (Human breast cancer) and A549 (Human lung cancer) cell lines. The in vitro anti tumor activity was screened by assessing tumor volume, viable and nonviable tumor cell count, tumor weight, hematological parameters and biochemical estimations by MTT Assay and Flow cytometry studies.
Data format	Analyzed data
Experimental factors	Leaf and heart wood was extracted in chloroform, water and methanol solvents to study their cytotoxic effect on human cancer cell lines and determine the extracts IC_50_ value.
Experimental features	The effect of leaf and heartwood extracts prepared in water and methanol on MCF-7 (Human breast cancer) and A549 (Human lung cancer) and Identification of compounds from *Caesalpinia sappan L.,* leaf and heartwood water and methanol extracts through LC–MS () and Docking studies against a BCl2 (B-cell lymphoma 2) protein which regulates the apoptosis.
Data source location	Yogi Vemana University campus green house facility (N 14°.473′, E 78°.710)
Data accessibility	Data are available within this article

**Value of the data**

• The data can be further explored to develop and design anticancer drugs for human Lung and breast cancer treatment from *Caesalpinia sappan* L. plant as a source for drugs [1], [2]
• These plant compounds can also be tried on other types of cancers for anticancer activity and compare with curing effect with the drugs currently in use, as plant based products are safer than synthetic drugs and with no side effects.

## 1 Data

**Fig. 1:**
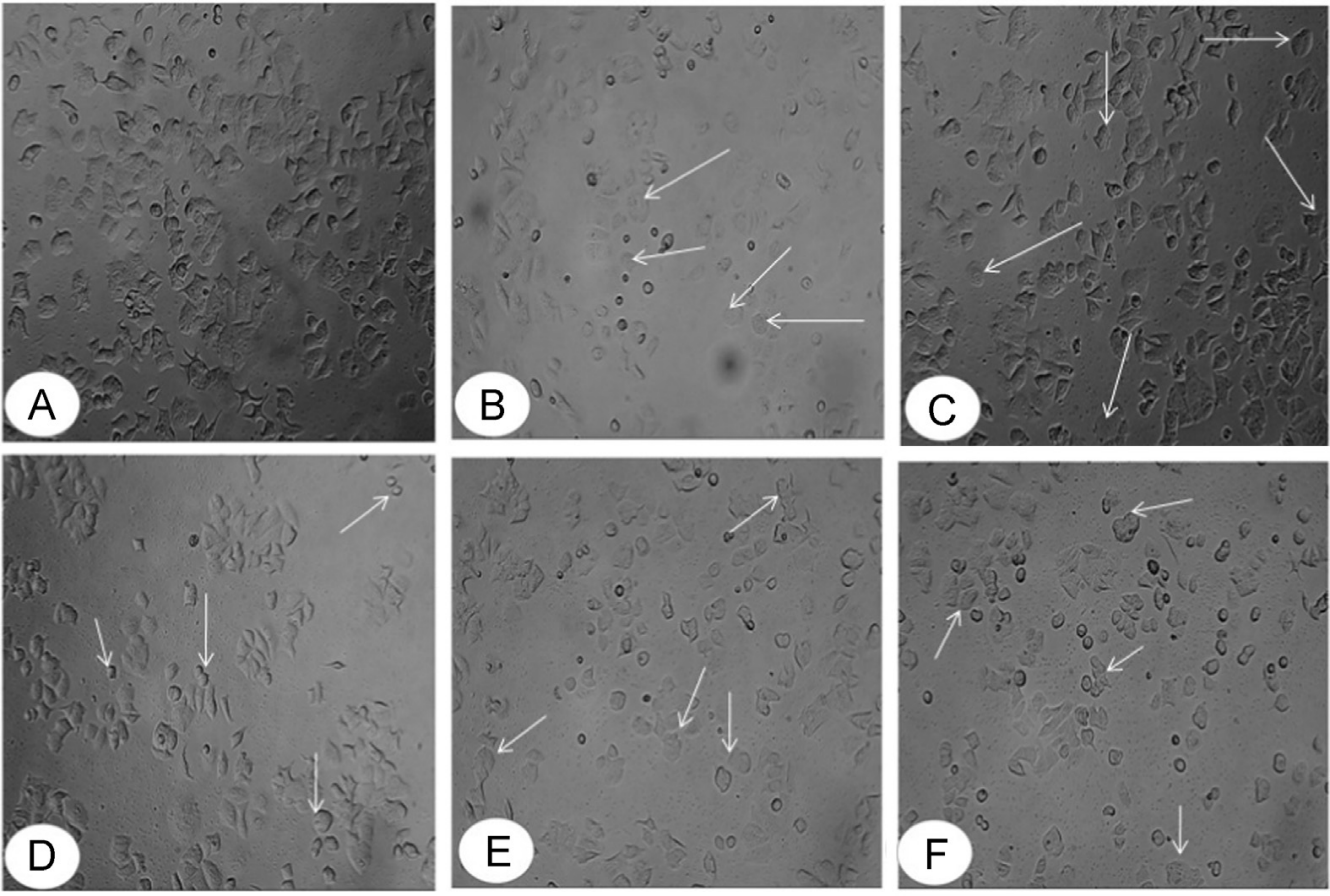
Morphological changes in cells of MCF-7 after treatment with heartwood methanol extracts of *Caesalpinia sappan L.* A: Untreated Cell lines, B: +Ve Control (Campotheterin −40 µM), C: –Ve Control (DMSO), D–F: *Caesalpinia sappan L.* Heartwood methanol extracts, D: 50 µg/ml, E: 150 µg/ml, F: 250 µg/ml.

**Fig. 2:**
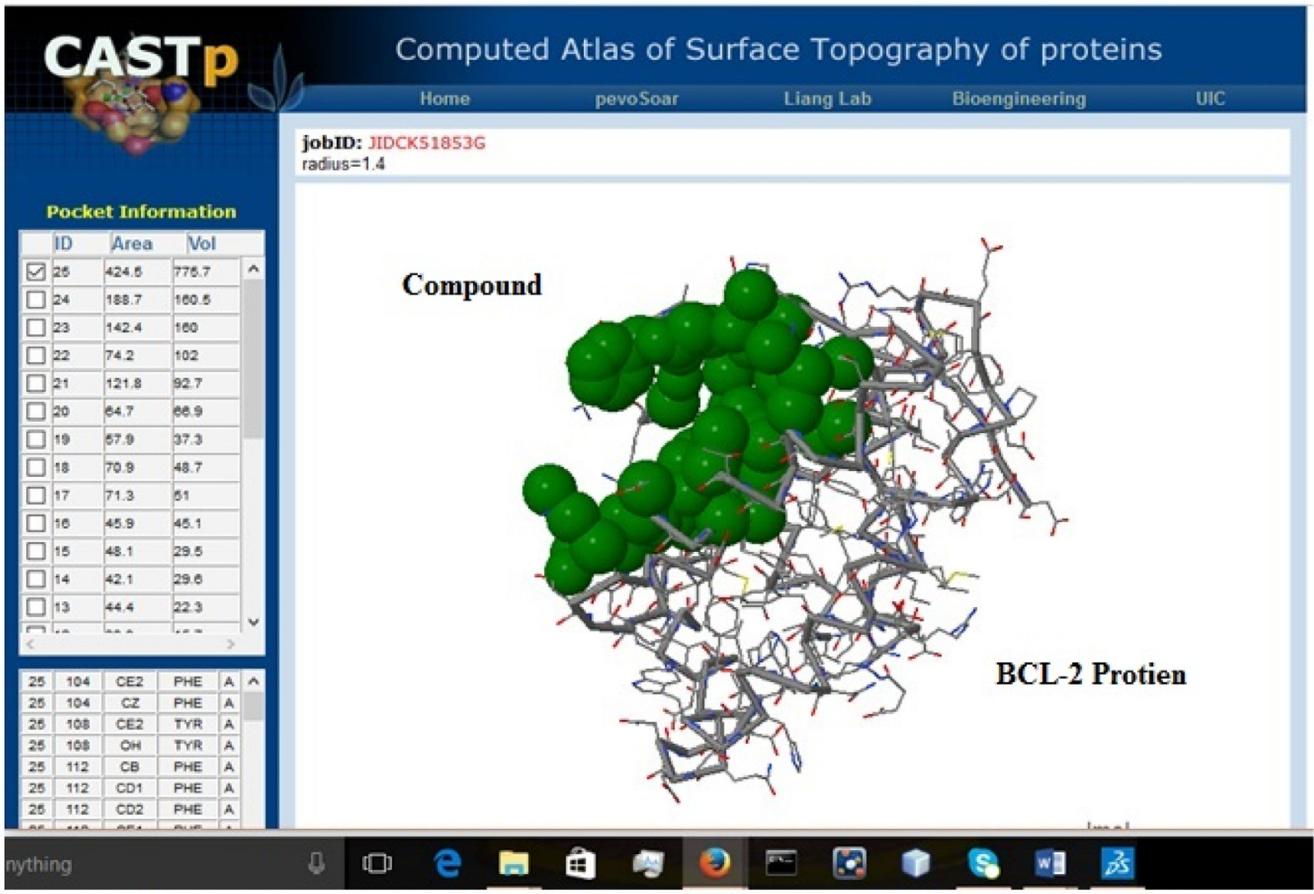
The 3D structure of Docked molecule (Brazilin A from *Caesalpinia sappan L.* heartwood extract) binding to receptors of BCL-2 protein, an anti apoptotic protein selected as target molecule.

**Fig. 3:**
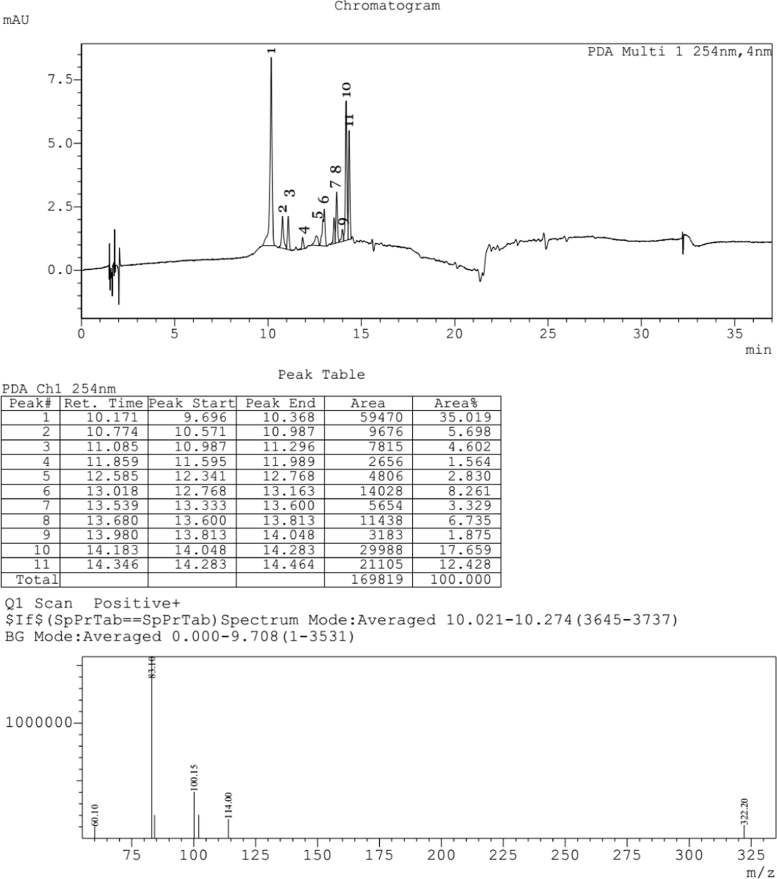
LCMS chromatogram of *Caesalpinia sappan L.* heartwood methanol extract. 10 µL of sample was loaded in eclipse XDB C18 column (150*4.6 mm and 5µ pore size), with 1.0 mL/min flow rate of Methanol:Water (80:20) as mobile phase. Mass spectra was performed by ESI (Electro spray Ionization), the formation of positive and negative ions occurs in high yield which is useful for determination of compounds.

**Fig. 4:**
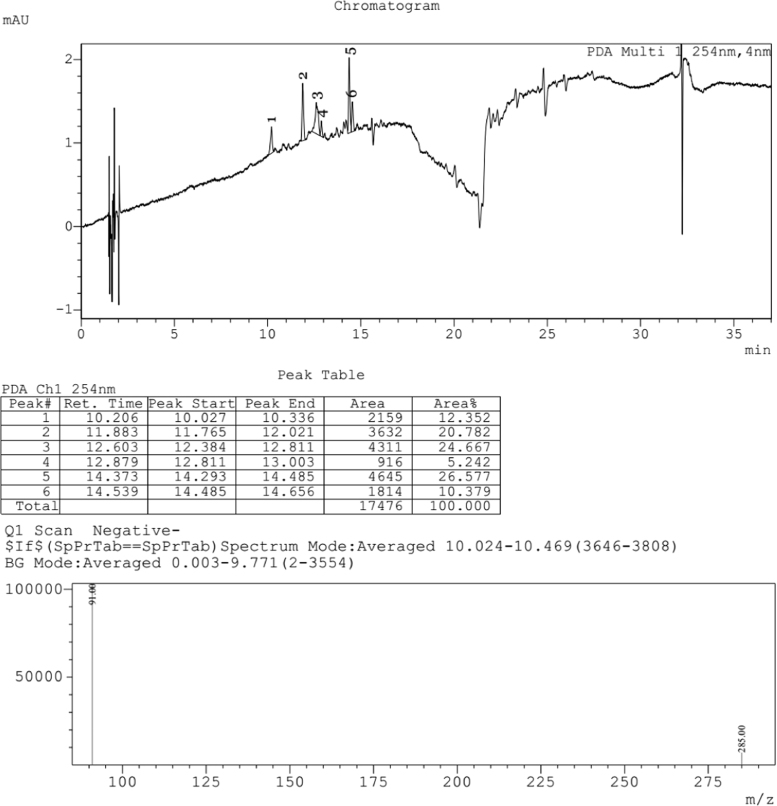
LCMS chromatogram of *Caesalpinia sappan L.* heartwood water extract. 10 µL of sample was loaded in eclipse XDB C18 column (150*4.6 mm and 5µ pore size), with 1.0 mL/min flow rate of Methanol: Water (80:20) as mobile phase. Mass spectra was performed by ESI (Electro spray Ionization), the formation of positive and negative ions occurs in high yield which is useful for determination of compounds.

**Fig. 5:**
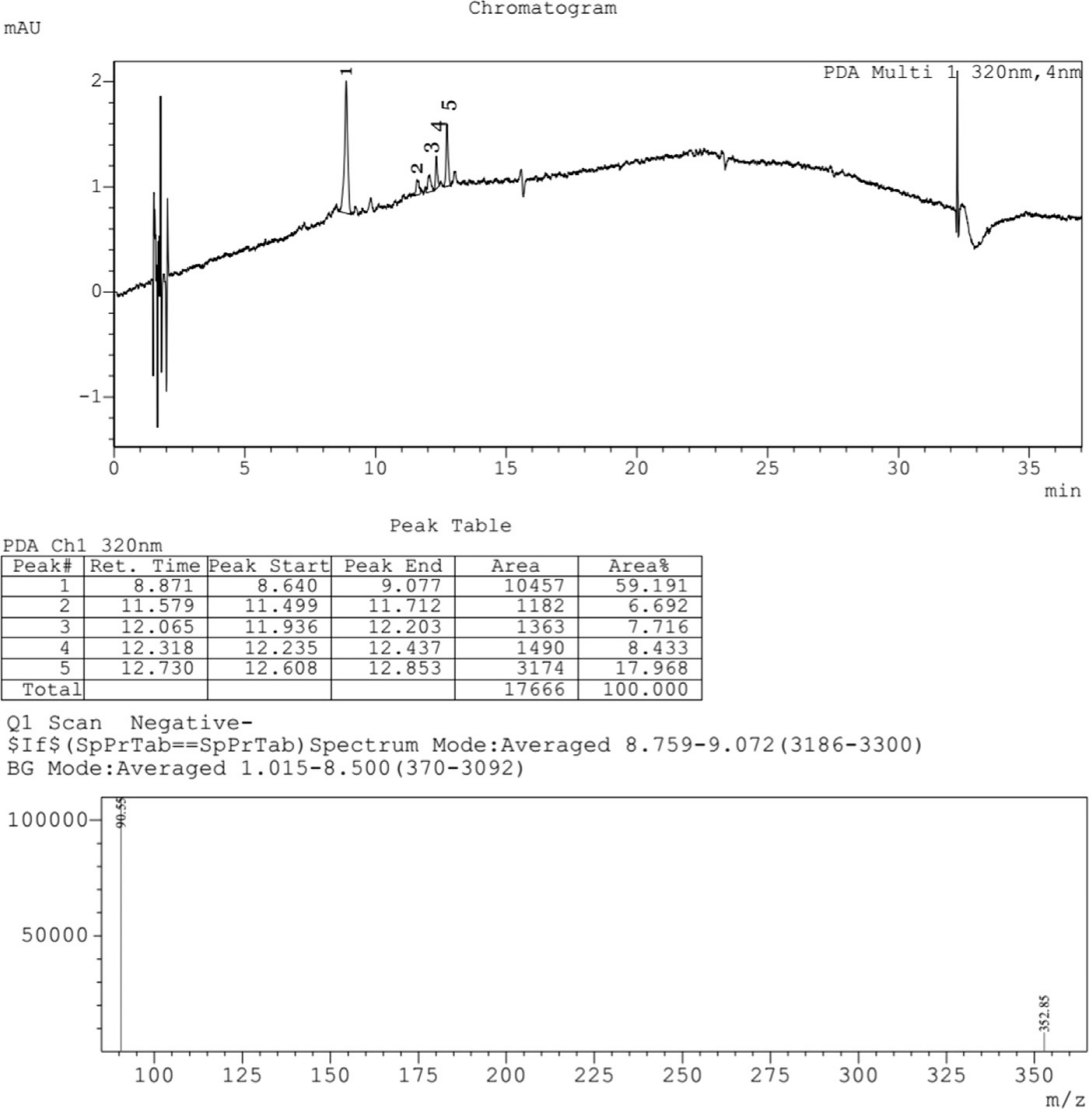
LCMS chromatogram of *Caesalpinia sappan L.* leaf methanol extract. 10 µL of sample was loaded in eclipse XDB C18 column (150*4.6 mm and 5µ pore size), with 1.0 mL/min flow rate of Methanol: Water (80:20) as mobile phase. Mass spectra was performed by ESI (Electro spray Ionization), the formation of positive and negative ions occurs in high yield which is useful for determination of compounds.

**Fig. 6:**
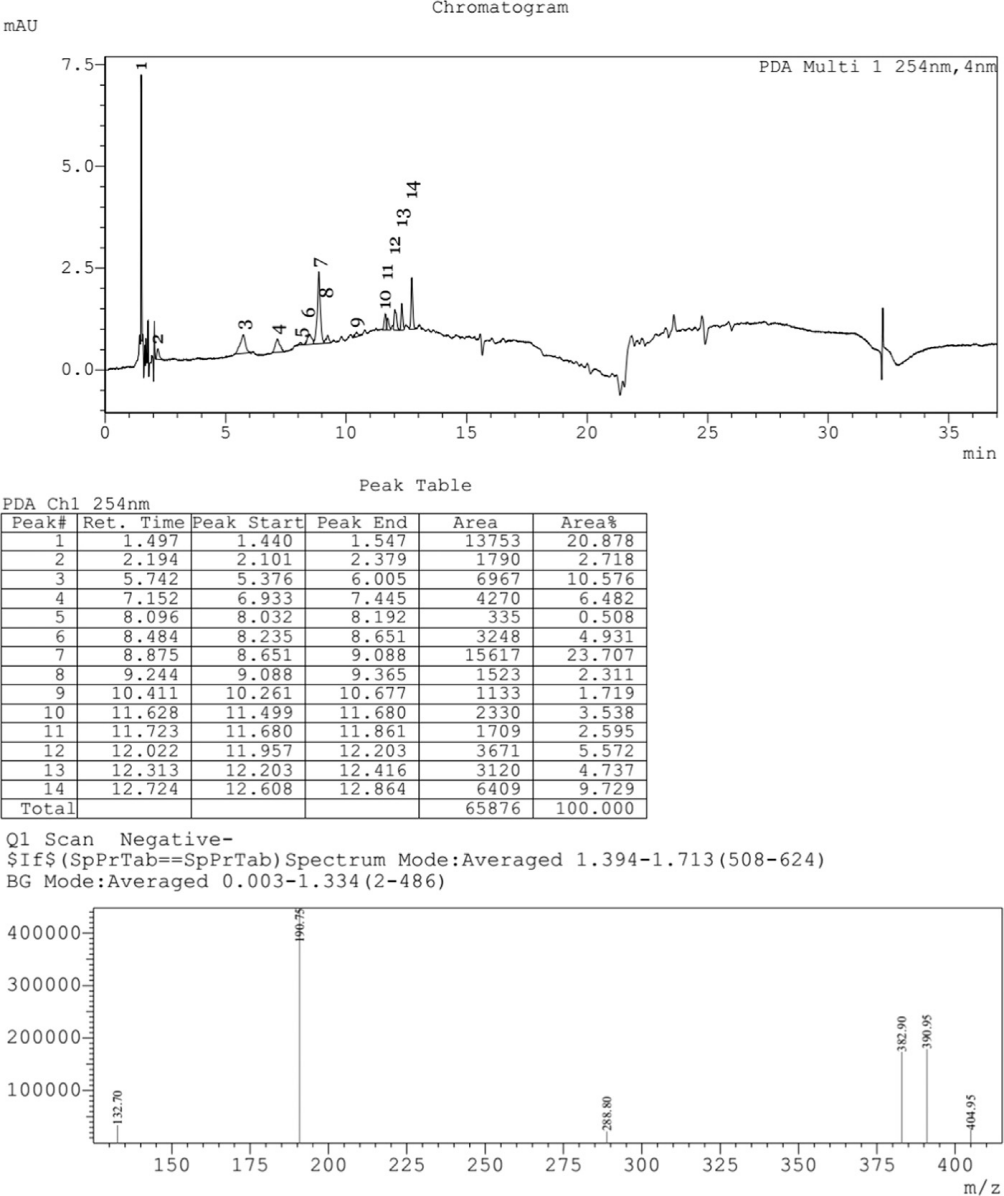
LCMS chromatogram of *Caesalpinia sappan L.* leaf water extract. 10 µL of sample was loaded in eclipse XDB C18 column (150*4.6 mm and 5µ pore size), with 1.0 mL/min flow rate of methanol:water (80:20) as mobile phase. Mass spectra was performed by ESI (Electro spray Ionization), the formation of positive and negative ions occurs in high yield which is useful for determination of compounds.

The Dataset in this study shows the potential of leaf and heart wood extracts (chloroform, methanol and water) of *Caesalpinia sappan L.* (Family: *Caesalpiniaceae L.)* as anti cancer agents(Fig. 1 and Table 1) which can be used further for drug development and designing in pharmaceutical industry. The Protein BCL-2 was used for carrying out docking studies (Fig. 2) with the compounds from leaf and heartwood (Fig. 3, Fig. 4, Fig. 5, Fig. 6) (Table 2).

**Table 1:** Effect of *Caesalpinia sappan L.* leaf (L) and heartwood (H) extracts prepared in chloroform, methanol and water on MCF-7 (Human breast cancer) and A549 (Human lung cancer) cells, Camptothecin is taken as positive control.

**Cell line**	**Plant sample**	**Camptothecin (50 uM)**	**50** **µg/ml**	**150** **µg/ml**	**250** **µg/ml**	**350** **µg/ml**	**450** **µg/ml**
**A549 Cell line**	L CHCL_3_	57.48±0.03	87.2±0.03	86.37±0.01	83.41±0.02	71.56±0.04	67.07±0.00
	LH_2_O	57.48±0.03	86.87±0.13	84.14±0.02	92.55±0.03	94.77±0.01	97.48±0.00
	L MET	57.48±0.03	101.52±0.00	89.50±0.01	89.34±0.07	86.00±0.04	84.23±0.02
	H H_2_O	57.48±0.03	81.06±0.29	87.69±0.00	88.35±0.00	88.06±0.03	96.82±0.06
	H Met	57.48±0.03	100.12±0.00	80.69±0.01	78.14±0.06	78.02±0.07	60.12±0.07
**MCF7 Cell line**	L CHCL_3_	56.32±0.02	98.84±0.07	102.17±0.01	108.63±0.06	112.24±0.01	102.64±0.01
	LH_2_O	56.32±0.02	105.10±0.00	104.76±0.00	102.24±0.02	97.82±0.03	96.39±0.14
	L MET	56.32±0.02	107.68±0.01	96.33±0.05	85.54±0.19	63.96±0.05	46.19±0.00
	H H_2_O	56.32±0.02	107.00±0.00	93.12±0.01	62.51±0.01	37.89±0.02	12.04±0.00
	H Met	56.32±0.02	101.15±0.01	52.31±0.00	4.01±0.00	2.31±0.00	1.22±0.01

**Table 2:** Binding energy of various secondary metabolites in methanolic extract of *Caesalpinia sappan L* with BCL-2 Protein by using auto dock software.

**S NO**	**Secondary metabolites**	**Binding energy (kcal/mol)**
**1.**	4-O-methylsappanol	−6.6
**2.**	Protosappanin B,	−6.9
**3.**	protosappanin A,	−7
**4.**	caesalpin J,	−6.6
**5.**	BrazilinA	−7
**6.**	BrazilinB	−7
**7.**	BrazilinC	−7
**8.**	Brazilein.	−6.9

## 2 Experimental design, materials and methods

### 2.1 Cell culture

The pure cultures of *MCF-7* (Breast cancer cell line) and *A549* ( Lung Cancer), were obtained From National Centre for Cell Science, Pune, Maharashtra state,India. The cells were grown and maintained in RPMI – 1640 media, supplemented with 10% v/v foetal bovine serum, sodium carbonate with 100 mg/l penicillin, 50 mg/l streptomycin to prevent the bacterial contamination and incubated at 37 °C in a humidified atmosphere having 5% CO_2_.

### 2.2 Anticancer assay

Soxhlet extraction method [3] was used for extraction of heart wood and powdered leaf sample of *Caesalpinia sappan L.* The cytotoxic activity of these extracts was tested against MCF7 and A549 cell lines and determined by MTT assay. This assay was performed in a 96-well culture plate according to a previously published protocol [4]. Percentage of viability was checked by calculating simulation index using the following formulae.

$$\mathrm{Stimulation\backslash index}=\frac{\mathrm{Absorbance\backslash with\backslash plant\backslash extract}}{\mathrm{Absorbance\backslash without\backslash plant\backslash extract}}$$

%ofviability=Stimulationindex×100

**Graph 1:**
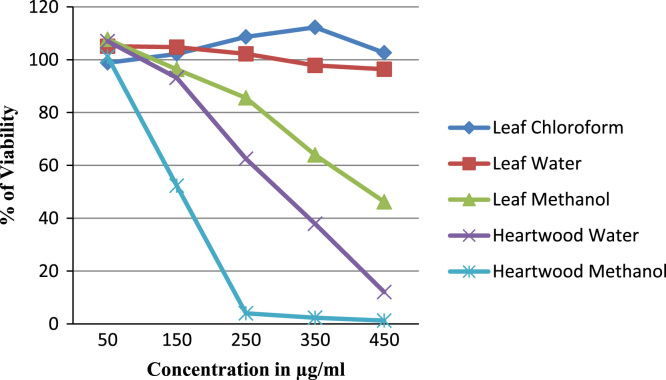
MTT analysis with different concentration of *Caesalpinia sappan L.* leaf and heart wood extracts in chloroform, water and methanol on MCF-7 (Human breast cancer) cell line. *L – leaf and H – heartwood.

**Graph 2:**
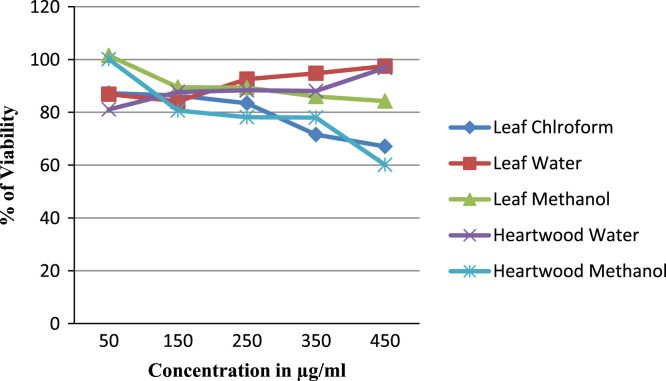
MTT analysis with different concentration of *Caesalpinia sappan L.* leaf and heart wood extracts in chloroform, water and methanol on A549 (Human lung cancer) cell line. *L – leaf and H – heartwood. .

The plant extract were subjected to LC–MS (SHIMADZU LCMS-2020) chromatography and UPLC–MS chromatography to identify the compounds in them (Fig. 3, Fig. 4, Fig. 5, Fig. 6). BCL-2 protein is an anti apoptotic protein selected as target molecule. The 3D structure of the compounds present in the sample was drawn in chemsketch tool. Further docking studies was carried out using auto dock [5] using bioactive molecule (Fig. 2) (Graph 1, Graph 2).

## Figures and Tables

**Fig. S1:**
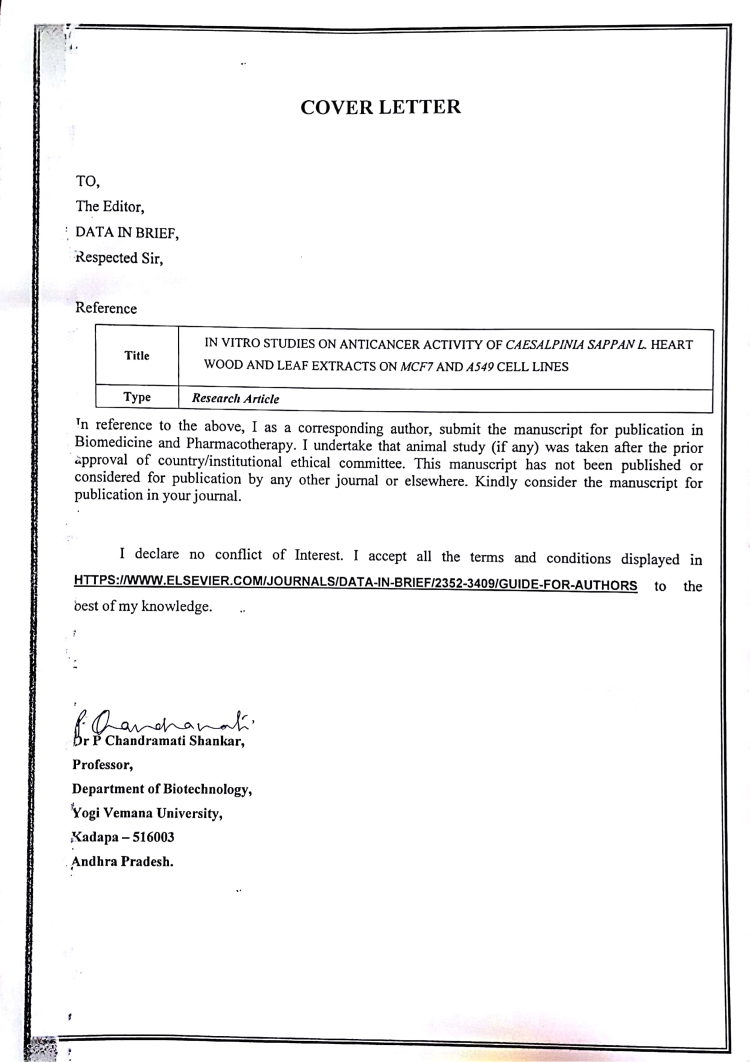


## References

[1] Ika Nurzijah (2017). Secang (*Caesalpinia sappan L*.) heartwood ethanolic extract shows activity as doxorubicin co-chemotherapeutic agent by apoptosis induction on T47D breast cancer cells. Indones. J. Cancer Chemoprev..

[2] Rahmawaty Rachmady (2017). Antiproliferative effect of secang heartwood ethanolic extract (*Caesalpinia sappan L*.) on HER2-positive breast cancer cells. Indones. J. Cancer Chemoprev..

[3] Naik Bukke Arunkumar, Hadi Fathima Nazneen, Shankar Produtur Chandramati (2015). Comparative study of in vitro antibacterial activity of leaves, bark, heart wood and seed extracts of *Caesalpinia sappan L.*. Asian Pac. J. Trop. Dis..

[4] Senthilraja P., Kathiresan K. (2015). in vitro cytotoxicity MTT assay in Vero, HepG2 and MCF-7 cell lines study of Marine Yeast. J. Appl. Pharm. Sci..

[5] Morris G.M., Huey R., Lindstrom W., Sanner M.F., Belew R.K., Goodsell D.S., Olson A.J. (2009). Autodock4 and AutoDockTools4: automated docking with selective receptor flexibility. J. Comput. Chem..

